# Treatment of a massive naproxen overdose with therapeutic plasma exchange in a dog

**DOI:** 10.1002/ccr3.2277

**Published:** 2019-06-28

**Authors:** Kathleen Kicera‐Temple, Leonel Londoño, Travis M. Lanaux, Gareth J. Buckley

**Affiliations:** ^1^ Department of Small Animal Clinical Sciences University of Florida Gainesville Florida USA

**Keywords:** acute kidney injury, non‐steroidal anti‐inflammatory drugs, therapeutic plasma exchange, toxicity

## Abstract

In comparison with other over‐the‐counter anti‐inflammatory drugs, naproxen has a longer half‐life in dogs and can lead to severe morbidity and mortality. This report describes the successful use of membrane‐based therapeutic plasma exchange after a massive ingestion of naproxen by a dog resulting in 86% reduction in plasma concentration.

## 1 INTRODUCTION

Naproxen is labeled as a traditional non‐steroidal anti‐inflammatory drug (NSAID) as it nonselectively inhibits cyclooxygenase type 1 (COX‐1) and cyclooxygenase type 2 (COX‐2) enzymes.^1^ Naproxen has a fivefold increase in inhibitory selectivity for COX‐1 compared to COX‐2 leading to a more pronounced morbidity when an overdose occurs in both human and veterinary patients.^2^, ^3^ It is further categorized as an acidic (vs nonacidic) NSAID with a pKa of 4.15, making it a weak acid.^4^, ^5^Acidic NSAIDs are known for having a high affinity for protein binding.^6^ Naproxen was found to be at least 99% protein bound in a canine experimental study performed by Frey et al evaluating the pharmacokinetics of naproxen.^5^, ^7^ The half‐life of naproxen in people ranges from 12 to 17 hours with a peak plasma concentration of 2‐4 hours and is almost entirely renally excreted.^1^, ^8^ For dogs, the half‐life is approximately 34‐74 hours with a peak plasma concentration between 0.5 and 3 hours.^7^ The half‐life of naproxen in dogs differs from that of people due to the different routes of excretion. In both dogs and people, naproxen is metabolized by the liver; however, in people, metabolites are primarily excreted by the kidney, whereas in dogs, the metabolites are partially excreted in bile and undergo extensive enterohepatic recirculation.^5^, ^7^ Naproxen has a low volume of distribution of 0.13 L/kg in dogs.^5^, ^7^ Based on the ASPCA Animal Poison Control Center (APCC), the estimated doses at which specific adverse effects occur are as follows: >5 mg/kg gastrointestinal signs, >10‐25 mg/kg acute kidney injury, and >50 mg/kg central nervous system signs.^9^

TPE is a type of extracorporeal blood purification therapy, which historically functions to treat patients with immune‐mediated diseases in both the veterinary and human medical fields.^10^, ^11^, ^12^ Recently, both the veterinary and human fields have found success in treating drug overdoses with TPE, specifically ibuprofen, meloxicam, and carprofen.^13^, ^14^, ^15^, ^16^ TPE is most efficient at removing highly protein‐bound drugs (>80% protein binding), with a low volume of distribution (ie, <0.2 L/kg).^7^, ^12^, ^13^, ^14^

## 2 CASE REPORT

A 9‐month‐old 24‐kg (52.8‐lb) neutered male mixed breed dog presented to a university teaching hospital after ingestion of up to 45 mg/kg (up to five 220 mg tablets) of naproxen, pens, paper products, and makeup a maximum of 3 hours prior to arrival. The patient presented bright, alert, and responsive with no remarkable findings on physical examination; the owners reported no clinical signs prior to presentation. Blood was collected for blood gas analysis, packed cell volume (PCV), and total solids (TS) (Table 1) which were within normal reference intervals. A urine sample was not obtained.

**Table 1:** Serial venous blood gas results in a dog receiving therapeutic plasma exchange for massive naproxen ingestion. All values reported in conventional units

Venous blood gas values and chemistry	Presentation	Post‐TPE	6 h post‐TPE
Packed cell volume (37%‐55%)	52%	51	41
Total plasma protein (52‐71 g/L [5.2‐7.1 g/dL])	6.0	4.0	4.4
Hematocrit (40.3%‐60.3%)	47%	51	44
pH (7.35‐7.42)	7.418	7.347	7.385
PCO_2_ (3.86‐5.60 kPa [29‐42 mm Hg])	34.4	34.3	39.0
PO_2_ (4.20‐8.56 pKa [31.6‐63.9 mm Hg])	47.1	57.5	39.8
HCO_3_ (17‐24 mmol/L [17‐24 mEq/L])	22.4	19.0	23.5
TCO2 (19‐26 mmol/L, [19‐26 mEq/L])	23.5	20.0	24.7
BEecf (−6.7 to 1.5 mmol/L [−6.7 to 1.5 mEq/L])	−2.4	−6.9	−1.8
Sodium (142‐152 mmol/L [142‐152 mEq/L])	148.9	146.0	147.0
Potassium (3.9‐5.1 mmol/L [3.9‐5.1 mEq/L])	4.05	3.53	4.10
Chloride (110‐124 mmol/L [110‐124 mEq/L])	113.2	116.8	115.1
Ionized calcium (1.16‐1.48 mmol/L [4.6‐5.9 mg/dL])	5.36	4.92	5.12
Magnesium (0.25‐0.41 mmol/L [0.13‐0.21 mEq/L])	1.0	0.86	0.9
Glucose (4.1‐7.0 mmol/L [68‐115 mg/dL])	91	152	93
Lactate (0.43‐2.1 mmol/L [3.8‐19 mg/dL])	12.6	5.5	2.5
BUN (2.9‐10.0 mmol/L [8‐28 mg/dL])	20	20	16
Creatinine (44‐150 µmol/L [0.5‐1.7 mg/dL])	1.4	1.1	1.2

Emesis was induced with apomorphine (0.04 mg/kg; Apomorphine USD; Letco) intravenously (IV) once, yielding a large volume of food, small pieces of plastic, a rubber pen grip, and a portion of clothing. No naproxen tablets were found in the vomitus. Due to the large dose of naproxen exposure, therapeutic plasma exchange was recommended. Treatment with IV lactated ringer's solution (Baxter Healthcare Corporation) was initiated at 140 mL/kg/d, and a single dose of maropitant (1 mg/kg; Cerenia; Zoetis Inc) was administered IV following successful emesis. The dog received 200 mL (8.3 mg/kg) of activated charcoal with sorbitol (ToxiBan suspension with sorbitol; Lloyd, Inc) once. Shortly thereafter, sedation with butorphanol (0.4 mg/kg IV; Torbugesic; Zoetis Inc), midazolam (0.3 mg/kg IV; Midazolam Injection, USP; Alvogen, Inc), and ketamine (5 mg/kg IV; Ketaset; Zoetis Inc) was administered. A 11.5F × 27 cm double‐lumen silicone dialysis catheter (Silicone two‐lumen hemodialysis catheter; MedComp) was placed in the right jugular vein using the Seldinger technique. TPE was performed using a PRISMA continuous renal replacement platform (Gambro Prismaflex^®^; Gambro Lundia AB) using a proprietary PRISMA TPE cartridge (Gambro TPE 2000^®^; Gambro Industries). Treatment started 4 hours after presentation and a maximum of seven hours after ingestion. A 112‐minute session was performed in which 1.6 L of plasma was exchanged. The average blood flow recorded was 107 mL/min (range of 100‐120 mL/min). Fluid replacement consisted of 1.2 L of lactated ringer's solution (Baxter Healthcare Corporation) and 400 mL of Hetastarch (Abbott Laboratories) premixed, administered throughout the TPE session. Anticoagulation was achieved with an initial bolus of 2400 units (100 U/kg) of unfractionated heparin (Heparin sodium injection 1000 USP Units/mL; Hospira Inc), followed by a continuous rate infusion of heparin at 1500 U/h to achieve an activated clotting time of 180‐250 seconds (reference range 60‐120 seconds).^17^, ^18^, ^19^ The patient's systolic, diastolic, and mean arterial blood pressures, heart rate and rhythm, and respiratory rate and effort were monitored throughout the session; all values remained normal. Following TPE, 20 mL/kg of fresh frozen plasma was administered IV over 1 hour without complication as a way of replacing coagulation factors and other plasma components removed during the plasma exchange. Blood gas analysis, PCV, and TS were performed immediately post‐TPE revealing a mild metabolic acidosis, hyperlactatemia, and hypoproteinemia; 6 hours post‐TPE, acidosis and hyperlactatemia had resolved and hypoproteinemia persisted (Table 1).

Blood and effluent samples were obtained at times 0, 15, 30, 60 and 112 minutes and again at 24 hours from the completion of the TPE session for naproxen level testing. Blood samples were collected in citrate tubes (BD Vacutainer Buff. Na Cirate) and centrifuged for 10 minutes at 5000 revolutions per minute after collection. The citrated plasma was then collected and transferred to sterile polypropylene Cryotubes (Nunc Cryotubes; Apogent). Effluent samples were collected directly into Cryotubes. All samples were frozen and stored at −80°C. Naproxen plasma and effluent concentrations were measured (Large Animal Analysis Laboratory, North Carolina State University) and values of percentage reduction of Naproxen from baseline are reported in Table 2.

**Table 2:** Serum naproxen concentration before and during, and 24 h after the initiation of therapeutic plasma exchange in a dog overdosed on naproxen

Time (min)	Plasma naproxen concentration (μg/mL)	Effluent naproxen concentration (μg/mL)	Percentage reduction of naproxen in plasma (%)
0	48.9	0	0
15	44.9	48.6	8.2
30	42.8	47	12.5
60	41.6	44.2	15
112	30.3	35.7	38
1440 (24 h)	7.10	n/a	85.5

Following TPE, the patient was continued on supportive care with IV lactated ringer's solution (Baxter Healthcare Corporation) at 140 mL/kg/d, activated charcoal without sorbitol (ToxiBan suspension; Lloyd, Inc) (10 mL/kg) every 8 hours for a total of two doses, misoprostol (Cytotec; Novel Laboratories, Inc) 100 μg (4 μg/kg) per os (PO) every 12 hours for a total of 12 doses, and IV pantoprazole (1 mg/kg; Protonix; AuroMedics Pharma LLC) every 12 hours. The patient was very hyperactive and regurgitated several times throughout the second day of hospitalization. The regurgitant was small in quantity and dark gray to black. He continued to have a normal appetite. The patient was started on metoclopramide (Reglan; Baxter Healthcare Corporation) at 2 mg/kg/d, sucralfate (Carafate; Greenstone LLC) 1 g (42 mg/kg) every 8 hours PO, and trazodone (Desyrel; Teva Pharmaceuticals USA, Inc) 100 mg (4 mg/kg) PO as needed every 8 hours for sedation. Thirty‐six hours following the TPE session, a chemistry panel was performed revealing mild panhypoproteinemia with a decreased total protein of 4.4 g/dL (56‐75 g/L [5.6‐7.5 g/dL]), albumin 2.2 g/dL (29‐38 g/L [2.9‐3.8 g/dL]), and globulin 2.2 g/dL (22‐42 g/L [2.2‐4.2 g/dL]), all other parameters within normal limits including BUN (22.4 mmol/L [8 mg/dL]) and creatinine (88.4 µmol/L [1.0 mg/dL]). The patient was discharged at the end of day 3 with instructions for additional misoprostol (Cytotec; Novel Laboratories, Inc) (4 μg/kg PO every 12 hours for 7 days), omeprazole (Omeprazole delayed release; Dexcel Pharma Technologies Ltd.) (0.83 mg/kg PO every 12 hours for 5 days), and sucralfate (Carafate; Greenstone LLC) (42 mg/kg PO every 8 hours for 3 days). On recheck 7 days later, the patient was clinically normal with no episodes of vomiting, regurgitation, melena, or inappetence. A chemistry panel was unremarkable with all values within normal reference intervals. A urinalysis revealed a urine specific gravity of 1.030 with an inactive sediment.

## 3 DISCUSSION

TPE is uniquely suited as a method of extracorporeal removal of drugs such as naproxen due to them being highly protein bound with a low volume of distribution and prolonged half‐life.^1^, ^7^, ^9^ Ultimately, a massive ingestion of 45 mg/kg of naproxen would be expected to cause acute kidney injury and potentially neurological signs which appeared to have been prevented by timely use of TPE in this case. One limitation of this report is that the lack of available renal biomarkers in clinical practice makes it difficult to rule out subclinical acute kidney injury secondary to the naproxen ingestion.

A previous study by Runkel et al^20^ found that a large portion of naproxen was excreted in the feces of dogs as compared to people where the majority of naproxen is excreted primarily via the kidneys.^5^, ^7^, ^8^ Oral absorption of naproxen in both dogs and people is rapid and is mainly hepatically metabolized in both species.^5^, ^7^ More recent evidence suggests that naproxen metabolites are mainly excreted in urine.^5^ At higher exposure doses, drug transporters and glucuronic acid conjugation pathway may become saturated, further contributing to the total drug exposure and thus a longer elimination half‐life. People and dogs have vastly different half‐lives of 12‐17 and 74 hours, respectively.^1^, ^5^, ^7^, ^8^ In dogs, metabolism of naproxen is thought to undergo extensive enterohepatic recirculation.^5^ Though not proven, this enterohepatic recirculation is suspected to contribute to the extended half‐life found in dogs as compared to people. Ultimately, this is also why naproxen in comparison with some of the other available NSAIDs can lead to worse clinical signs at lower ingested doses than other available veterinary and human NSAID products. Because even low doses of naproxen ingestion can cause significant morbidity in dogs in a case such as this where the ingested dose was very high, the benefits of extracorporeal removal of the drug likely outweighed the risks of TPE. In this case, the mild metabolic acidosis and hyperlactatemia observed immediately following TPE resolved 6 hours later after the patient received a transfusion of fresh frozen plasma and IV fluid therapy. The hypoproteinemia that the patient developed never leads to clinical consequences.

Twenty‐four hours after the initiation of TPE, the percentage reduction in plasma naproxen was 86% from a starting plasma naproxen concentration of 48.90 μg/mL (0.0489 g/L). Factors that may have affected the plasma naproxen concentrations from decreasing further at the 112‐minute mark include active absorption from the GI tract during TPE and redistribution from tissues to systemic circulation, the significance of which cannot be accounted for. Based on the half‐life of naproxen being 74 hours, endogenous clearance would predict plasma concentration at 24 hours of 38.9 μg/mL, which is only a 20% reduction from the time of initiation of TPE. The endogenous elimination half‐life calculated from the end of TPE until the 24‐hour time point in this patient is 10.55 hours. Based on current guidelines, a 1.5 plasma volume exchange aims to remove 78% of the target substance while a 2.0 plasma volume exchange only removes 86% (8% increase), which is considered clinically irrelevant, this has been observed in other cases of NSAID removal with TPE.^14^, ^15^, ^16^ This case report demonstrates that TPE in combination with oral administration of activated charcoal and additional supportive care can be effective in the treatment of high doses of naproxen ingestion.

## 4 CONCLUSION

Current indications for the use of TPE in acute intoxications include toxins with high degree of protein binding, low volume of distribution, and the potential for high morbidity and mortality. The dog in this case report ingested a potentially fatal dose of naproxen, a drug fitting the profile described above, without developing significant clinical signs, suggesting that TPE could be used as first‐line therapy for treatment of this intoxication in dogs. Further investigation is necessary to optimize the prescription for TPE, particularly assessing optimal plasma exchange volume and the use of multiple TPE sessions to increase the efficiency of toxin removal.

## CONFLICT OF INTEREST

The authors report no conflicts of interest for this case report.

## AUTHOR CONTRIBUTIONS

KK‐T: collected the data, analyzed and interpreted the data, and drafted and prepared the manuscript. LL: conceived and designed the study, analyzed and interpreted the data, drafted and prepared the manuscript, and critically revised the manuscript. TML: collected the data and critically revised the manuscript. GJB: analyzed and interpreted the data and critically revised the manuscript.
